# Age at Smoking Initiation and Prevalence of Cigarette Use Among Youths in Sub-Saharan Africa, 2014-2017

**DOI:** 10.1001/jamanetworkopen.2021.8060

**Published:** 2021-05-07

**Authors:** Onyema Greg Chido-Amajuoyi, Patrick Fueta, Dale Mantey

**Affiliations:** 1 Division of Cancer Prevention and Population Sciences, Department of Epidemiology, The University of Texas MD Anderson Cancer Center, Houston; 2 Department of Pediatrics, Texas A&M University/Driscoll Children’s Hospital, Corpus Christi; 3 Department of Health Promotion and Behavioral Sciences, The University of Texas School of Public Health, Austin

## Abstract

This cross-sectional study examines age at smoking initiation, prevalence of cigarette use, and prevalence of exposure to smoking-related risk factors among youths in sub-Saharan Africa.

## Introduction

The 2020 World No Tobacco Day campaign was focused on promoting measures to protect children and young people from exploitation by the tobacco industry. This comes as more than 40 million adolescents aged 13 to 15 years are reported to have already initiated tobacco use.^1^

The African continent has one of the lowest regional tobacco use rates and is generally considered to be at subepidemic levels, with lower smoking intensities compared with other parts of the world.^2^ However, evidence points to increasingly aggressive tobacco industry marketing in Africa, with a substantial proportion of these efforts targeted at youth.^3,4^ With increasing tobacco industry activity in this region, African countries are projected to progress to a tobacco epidemic by 2040.^2^ This study aimed to describe the prevalence of exposure to smoking-related risk factors, susceptibility to smoke, cigarette smoking, and age at smoking initiation among adolescents in 9 sub-Saharan African nations.

## Methods

This cross-sectional study analyzed data from the Global Youth Tobacco Surveys (GYTS) from 2014 to 2017 for Cameroon, Gabon, Comoros, Seychelles, Mauritius, Tanzania, Gambia, Ghana, and Sierra Leone. GYTS is a school-based survey designed to monitor behaviors and risk factors associated with adolescent tobacco use. The GYTS uses a 2-stage cluster sample to derive nationally representative samples for each nation.^5^ This study followed the Strengthening the Reporting of Observational Studies in Epidemiology (STROBE) reporting guideline. GYTS data are deidentified and publicly available, hence institutional review board approval was not required in accordance with 45 CFR §46. Passive parental consent was used for the GYTS. Parents received a notification form or letter prior to the survey and were allowed to opt out.

Weighted prevalence and 95% CIs were estimated for secondhand smoke exposure, parental and peer smoking (social exposure), and protobacco and antitobacco advertisement exposure. Prevalence of ever and current cigarette use and susceptibility to smoking initiation among never smokers was calculated, as well as the distribution of age at smoking initiation. A detailed description of measures is provided in an eAppendix in the Supplement. χ^2^ tests were used to examine for differences in cigarette use, as well as susceptibility to smoking initiation by sex. Statistical significance was determined at *P* < .05. Analyses were performed using Stata version 14.2 (StataCorp) from September 1 to September 28, 2021.

## Results

The overall sample (N = 38 313) represented a weighted population of 5 488 250 youths. Of this sample, 50.5% (20 677, unweighted) were female youths, with mean (SD) age of 14.3 (1.4) years. Secondhand smoking exposure at home ranged from 16.0% (95% CI, 13.4%-19.0%) in Tanzania to 33.8% (95% CI, 31.1%-36.5%) in Gambia (Table 1). In Seychelles, 68.6% (95% CI, 63.7%-73.2%) of youths were exposed to smoking by school peers. Protobacco advertising was most prevalent on television and as high as 64.5% (95% CI, 55.2%-72.8%) in Cameroon. Antismoking advertisement exposure were mostly via mass media (69.3% [95% CI, 66.8%-71.7%] in Gabon) and cigarette packs (62.3% [57.4%-67.0%] in Cameroon).

**Table 1.** Exposure to Smoking-Related Risk Factors and Antismoking Advertisements Among Adolescents in Sub-Saharan Africa: Global Youth Tobacco Survey 2014 to 2017

Exposures	Youths, Weighted % (95% CI)								
	2014		2015		2016		2017		
	Cameroon (n = 2691)	Gabon (n = 1654)	Comoros (n = 2511)	Seychelles (n = 2233)	Mauritius (n = 3893)	Tanzania (n = 3543)	Gambia (n = 11 035)	Ghana (n = 4975)	Sierra Leone (n = 5778)
Secondhand smoke^a^									
At home	27.9 (23.8-32.4)	31.9 (28.5-35.4)	26.3 (22.6-30.5)	30.1 (27.9-32.4)	28.7 (25.0-32.8)	16.0 (13.4-19.0)	33.8 (31.1-36.5)	20.4 (17.6-23.5)	25.7 (22.7-29.1)
Public (indoor)	44.1 (34.8-53.9)	59.6 (26.3-62.9)	43.2 (36.7-50.1)	39.5 (36.7-42.4)	44.0 (40.8-47.3)	33.7 (30.1-37.5)	62.1 (59.1-64.9)	37.2 (33.0-41.6)	42.4 (37.7-47.2)
Public (outdoor)	48.7 (38.1-59.5)	59.0 (54.4-63.4)	42.0 (36.9-47.2)	49.9 (46.9-52.8)	53.1 (50.1-56.1)	37.7 (34.0-41.7)	56.8 (54.1-59.5)	38.5 (35.2-42.0)	44.7 (39.4-50.1)
Social									
Parents	NA	NA	NA	31.1 (28.9-33.4)	31.8 (27.9-35.9)	8.9 (6.8-11.1)	NA	NA	NA
Friends	NA	NA	NA	53.5 (49.1-57.9)	49.0 (42.4-55.5)	7.9 (6.4-9.7)	NA	NA	NA
School peers	NA	NA	NA	68.6 (63.7-73.2)	62.0 (53.8-69.5)	11.2 (8.6-14.3)	NA	NA	NA
Protobacco advertisement^b^									
Television	64.5 (55.2-72.8)	58.4 (55.7-61.0)	44.2 (39.6-48.9)	60.2 (57.8-62.7)	55.2 (52.4-58.1)	39 (33.9-44.5)	49.3 (46.3-52.3)	48.2 (43.8-52.7)	48.7 (42.9-54.5)
Retail point of sale	25.1 (19.8-31.2)	20.1 (18.2-22.1)	18.5 (16.8-20.3)	17.3 (15.4-19.3)	14.8 (12.5-17.4)	26.7 (24.0-29.6)	15.1 (13.3-17.1)	18.3 (15.9-20.9)	30.8 (25.3-36.8)
Branded products	12.4 (9.5-16.2)	6.4 (5.1-8.1)	11.0 (9.2-13.1)	13.5 (11.9-15.3)	12.8 (10.7-15.3)	6.3 (5.3-7.4)	10.0 (8.9-11.1)	7.5 (6.2-8.9)	22.0 (18.3-26.2)
Free products	7.6 (4.5-12.4)	3.5 (2.6-4.6)	12.5 (10.2-15.3)	6.2 (5.0-7.6)	4.4 (3.2-6.1)	3.9 (3.2-4.8)	6.0 (5.2-7.0)	6.5 (5.0-8.4)	9.7 (7.7-12.2)
Antitobacco advertisement^c^									
Media	67.5 (61.1-73.2)	69.3 (66.8-71.7)	54.4 (51.0-57.7)	57.5 (54.8-60.2)	66.0 (61.4-70.3)	54.9 (49.0-60.6)	42.8 (40.3-45.4)	48.8 (44.7-52.9)	54.3 (49.4-59.1)
Events	24.8 (20.2-30.2)	22.7 (20.4-25.2)	22.4 (20.0-24.9)	23.6 (21.7-25.7)	29.8 (25.2-34.9)	27.7 (25.2-30.2)	24.8 (22.2-27.6)	25.6 (22.0-29.5)	31.8 (27.0-37.0)
Cigarette packaging	62.3 (57.4-67.0)	25.0 (21.6-28.6)	35.9 (33.2-38.7)	50.9 (48.0-53.9)	62.7 (59.1-66.1)	43.3 (38.3-48.4)	57.6 (54.4-60.6)	41.3 (37.3-45.3)	48.1 (42.4-53.9)

Abbreviation: NA, not available.

^a^ Sites for secondhand smoke exposure considered were (1) at home, (2) in an indoor public place, and (3) in an outdoor public place.

^b^ Channels of protobacco advertising considered were (1) television (TV, videos, or movies), (2) point of sale (eg, in kiosks, malls, supermarkets), (3) offered free tobacco product from tobacco company representative, and (4) ownership of a tobacco company branded material.

^c^ Channels of antitobacco advertising considered were (1) via any media (eg, television, movies, radio, internet, billboards, print), (2) at public events (eg, sports event), and (3) health warnings on cigarette packages.

Weighted prevalence of ever cigarette use ranged from 5.4% (95% CI, 3.8%-7.6%) in Tanzania to 36.4% (95% CI, 32.3%-40.8%) in Seychelles (Table 2). Current cigarette use ranged from 1.0% (95% CI, 0.6%-1.8%) in Tanzania to 15.4% (95% CI, 13.1% 18.1%) in Seychelles. Susceptibility to smoking initiation ranged from 7.3% (95% CI, 5.3%-10.0%) in Tanzania to 28.9% (95% CI, 25.2%-33.0%) in Sierra Leone. In all countries, cigarette smoking prevalence was greater among male youths compared with female youths (eg, in Gabon: 40.8% [35.1%-46.7%] of male youths had ever smoked vs 17.1% [11.8%-24.0%] of female youths; *P* < .001). Among ever smokers, more than 20% of youths started smoking by age 8 years in Tanzania, Ghana, and Sierra Leone. In all study countries, more than 75% of youths who smoked initiated smoking before age 15 years (Table 2).

**Table 2.** Cigarette Use–Related Characteristics of Adolescents in Sub-Saharan Africa: Global Youth Tobacco Survey 2014 to 2017

Cigarette use–related characteristics	Youths, Weighted % (95% CI)								
	2014		2015		2016		2017		
	Cameroon (n = 2691)	Gabon (n = 1654)	Comoros (n = 2511)	Seychelles (n = 2233)	Mauritius (n = 3893)	Tanzania (n = 3543)	Gambia (n = 11 035)	Ghana (n = 4975)	Sierra Leone (n = 5778)
Ever users^a^									
Total	16.1 (11.3-22.5)	31.4 (27.9-35.0)	14.1 (10.4-18.9)	36.4 (32.3-40.8)	28.5 (22.9-34.8)	5.4 (3.8-7.6)	19.8 (17.6-22.2)	7.3 (5.8-9.3)	11.6 (8.8-15.2)
Male	21.9 (15.0-30.8)	39.6 (34.4-45.1)	21.3 (17.3-25.9)	40.0 (35.6-44.5)	40.8 (35.1-46.7)	8.4 (5.8-12.1)	32.4 (28.8-36.2)	9.4 (7.2-12.0)	14.4 (11.7-17.6)
Female	9.6 (7.4-12.4)	23.6 (19.6-28.1)	7.6 (4.1-13.7)	33.1 (28.3-38.3)	17.1 (11.8-24.0)	2.6 (1.7-4.1)	8.6 (7.1-10.4)	5.3 (3.5-7.9)	8.8 (5.2-14.5)
*P* value	<.001	<.001	<.001	.003	<.001	<.001	<.001	.01	.03
Current users^b^									
Total	6.8 (4.1-11.0)	9.0 (7.6-10.7)	7.4 (5.6-9.6)	15.4 (13.1-18.1)	15.3 (10.1-21.0)	1.0 (0.6-1.8)	8.5 (7.3-10.0)	2.8 (2.0-3.9)	3.8 (2.7-5.2)
Male	9.9 (6.0-16.1)	13.6 (11.9-15.5)	12.1 (9.6-15.2)	20.3 (17.2-23.9)	23.6 (17.5-31.1)	1.7 (0.9-3.0)	14.5 (12.2-17.1)	3.3 (2.5-4.3)	6.3 (4.6-8.5)
Female	3.2 (2.0-5.3)	4.7 (3.0-7.2)	3.0 (1.6-5.6)	10.8 (8.7-13.4)	7.5 (4.6-12.0)	0.04 (0.00-0.012)	3.3 (2.5-4.3)	2.4 (1.3-4.3)	1.2 (0.7-2.2)
*P* value	<.001	<.001	<.001	<.001	<.001	.02	<.001	.24	<.001
Susceptibility to smoking^c^^,^^d^									
Total	27.7 (20.3-36.6)	20.1 (17.1-23.5)	28.0 (23.4-33.2)	19.5 (16.8-22.6)	11.5 (9.7-13.6)	7.3 (5.3-10.0)	24.5 (21.5-27.7)	22.8 (19.8-26.1)	28.9 (25.2-33.0)
Male	28.2 (20.4-37.6)	17.7 (14.1-22.0)	29.0 (24.5-33.9)	22.0 (18.5-26.0)	14.2 (10.4-19.0)	7.0 (4.9-9.9)	25.8 (22.2-29.7)	22.1 (19.5-25.0)	30.6 (27.0-34.4)
Female	27.2 (19.3-36.9)	21.9 (17.6-26.9)	27.3 (21.9-33.4)	17.4 (14.1-21.3)	9.8 (7.7-12.3)	7.8 (5.0-11.5)	23.6 (20.4-27.2)	23.5 (19.7-27.8)	27.3 (22.7-32.6)
*P* value	.73	.16	.45	.047	.06	.70	.23	.33	.12
Age at initiation^e^									
7 or less	14.0	10.1	11.1	5.6	4.9	24.1	15.2	22.4	22.4
8-9 y	21.0	7.3	12.4	8.1	7.2	12.4	13.3	19.7	16.9
10-11 y	19.5	15.3	14.6	14.8	14.0	15.5	18.3	23.4	18.1
12-13 y	17.2	17.9	21.4	34.5	43.7	34.3	21.1	19.7	18.4
14-15 y	17.2	29.0	25.4	32.4	26.5	12.2	20.5	12.8	14.9
16+ y	11.1	20.5	15.1	4.7	4.8	1.6	11.7	2.0	9.4

^a^ Respondents were considered ever users if they reported ever trying a cigarette, even one or two puffs, in their lifetime.

^b^ Respondents were considered current users if they reported use of cigarettes in the past 30 days.

^c^ Subpopulation estimation among never smokers.

^d^ Respondents were considered susceptible to smoking if they provided responses other than “definitely not” to the survey questions: “If one of your best friends gives you a cigarette, would you smoke it?” and “At any time during the next 12 months do you think you will smoke a cigarette?”

^e^ Subpopulation estimation among ever smokers.

## Discussion

In sub-Saharan African nations, ever and current cigarette smoking were as high as 36.4% and 15.4%, respectively, with use being significantly higher among boys. Equally concerning is the widespread early age of onset of smoking in this region; as well as the fact that despite lower smoking rates, girls seemed to be as susceptible to smoking initiation as boys.

Findings call for stricter enforcement of the World Health Organization Framework Convention on Tobacco Control–compliant policies throughout the region and conscious efforts geared to protect children from tobacco industry exploitation. For example, despite comprehensive bans on tobacco advertising, promotion and sponsorship,^6^ Seychelles and Mauritius have the highest smoking rates reported in this study. Specific adolescent-targeted interventions include enforcing bans on sales of tobacco products to minors and adding smoking prevention courses to the curricula of schools throughout sub-Saharan Africa. Study limitations include the fact that study data are self-reported and prone to recall and social desirability bias.

## Notes

Supplement. **eAppendix.** Measures eReferences.
