# Supplementary material for: Age at Smoking Initiation and Prevalence of Cigarette Use Among Youths in Sub-Saharan Africa, 2014-2017
Source: JAMA Netw Open. 2021 May 7;4(5):e218060. doi: 10.1001/jamanetworkopen.2021.8060 (PMC8105748; doi:10.1001/jamanetworkopen.2021.8060)
Supplement: Supplement. — eAppendix. Measures eReferences. [file jamanetwopen-e218060-s001.pdf]

Supplemental Online Content

Chido-Amajuoyi OG, Fueta P, Mantey D. Age at smoking initiation and prevalence of cigarette use among youths in Sub-Saharan Africa, 2014-2017. *JAMA Netw Open*. 2021;4(5):e218060. doi:10.1001/jamanetworkopen.2021.8060

eAppendix. Measures

eReferences

This supplemental material has been provided by the authors to give readers additional information about their work.

© 2021 Chido-Amajuoyi OG et al. *JAMA Network Open.*

eAppendix. Measures

Ever cigarette use: Respondents were considered ever users if they reported ever trying a cigarette, even one or two puffs, in their lifetime. Specifically, respondents were asked, 'Have you ever tried or experimented with cigarette smoking, even one or two puffs?' with those reporting 'yes' categorized as "ever users".

Current cigarette use: Respondents were considered current users if they reported use of cigarettes in the past 30days, and those that had not were considered non-users. Specifically, respondents were asked, 'During the past 30days, on how many days did you smoke cigarettes?' with those reporting one or more days categorized as "current users".

Susceptibility to use cigarette: Considered among never users of cigarettes. This measure employed a two-item scale established by Pierce et al.[1] Questions used to measure susceptibility were: 'If one of your best friends gives you a cigarette, would you smoke it?' and 'At any time during the next 12 months do you think you will smoke a cigarette?' Possible responses for these questions were 'definitely yes', 'probably yes', 'probably not' and 'definitely not'. Respondents who selected responses other than "definitely not" for either question were considered susceptible to smoking cigarettes.

Pro-Tobacco Advertising Exposure: This was derived based on responses to the following questions: (1) "during the past 30-days, did you see any people using tobacco on TV, in videos, or in movies?" (2) during the past 30-days, did you see any advertisements or promotions for tobacco products at points of sale (such as stores, shops, kiosks, super markets, etc.)?"; (3) "do you have something (for example, t-shirt, pen, backpack) with a tobacco product brand logo on it?"; and (4) "Has a person working for a tobacco company ever offered you a free tobacco product?" Possible responses for each question were "yes" (coded as 1) and "no" (coded as 0).

Anti-Tobacco Advertising Exposure: This was derived based on responses to the following questions: (1) "During the past 30 days, did you see or hear any anti-tobacco media messages on television, radio, internet, billboards, posters, newspapers, magazines, or movies?" (2) During the past 30 days, did you see or hear any anti-tobacco messages at sports events, fairs, concerts, or community events, or social gatherings?"; (3) "During the past 30 days, did you see any health warnings on cigarette packages?" Possible responses for each question were "yes" (coded as 1) and "no" (coded as 0).

Secondhand smoke exposure: This was based on responses to the following questions: (1) "during the past 7-days, on how many days has anyone smoked inside your home, in your presence?"; (2) "during the past 7-days, on how many days has anyone smoked in your presence, inside any enclosed public place, other than your home (such as school, shops, restaurants, super markets, movie theaters, cyber cafes?"; and (3) "during the past 7-days, on how many days has anyone smoked in your presence, at any outdoor public place (such as playgrounds, sidewalks, entrances to buildings, bus stops, beaches)?" Participants who provided a response other than "0 days" to any of the three questions were considered exposed to secondhand smoke.

eReferences

1. Pierce, J. P., Choi, W. S., Gilpin, E. A., Farkas, A. J., & Merritt, R. K. (1996). Validation of susceptibility as a predictor of which adolescents take up smoking in the United States. Health psychology, 15(5), 355.

© 2021 Chido-Amajuoyi OG et al. *JAMA Network Open.*
